# Food Insecurity as a Barrier to Sustained Antiretroviral Therapy Adherence in Uganda

**DOI:** 10.1371/journal.pone.0010340

**Published:** 2010-04-28

**Authors:** Sheri D. Weiser, David M. Tuller, Edward A. Frongillo, Jude Senkungu, Nozmu Mukiibi, David R. Bangsberg

**Affiliations:** 1 Division of HIV/AIDS, San Francisco General Hospital, San Francisco, California, United States of America; 2 Center for AIDS Prevention Studies, University of California San Francisco, San Francisco, California, United States of America; 3 School of Public Health, University of California, Berkeley, California, United States of America; 4 Department of Health Promotion, Education, and Behavior, Arnold School of Public Health, University of South Carolina, Columbia, South Carolina, United States of America; 5 Mbarara University of Science and Technology, Mbarara, Uganda; 6 Ragon Institute of Massachusetts General Hospital, Massachusetts Institute of Technology and Harvard, Boston, Massachusetts, United States of America; 7 Massachusetts General Hospital Center for Global Health, Harvard Medical School, Cambridge, Massachusetts, United States of America; 8 Harvard Initiative for Global Health, Boston, Massachusetts, United States of America; Institute for Tropical Medicine Antwerp, Belgium

## Abstract

**Background:**

Food insecurity is emerging as an important barrier to antiretroviral (ARV) adherence in sub-Saharan Africa and elsewhere, but little is known about the mechanisms through which food insecurity leads to ARV non-adherence and treatment interruptions.

**Methodology:**

We conducted in-depth, open-ended interviews with 47 individuals (30 women, 17 men) living with HIV/AIDS recruited from AIDS treatment programs in Mbarara and Kampala, Uganda to understand how food insecurity interferes with ARV therapy regimens. Interviews were transcribed, coded for key themes, and analyzed using grounded theory.

**Findings:**

Food insecurity was common and an important barrier to accessing medical care and ARV adherence. Five mechanisms emerged for how food insecurity can contribute to ARV non-adherence and treatment interruptions or to postponing ARV initiation: 1) ARVs increased appetite and led to intolerable hunger in the absence of food; 2) Side effects of ARVs were exacerbated in the absence of food; 3) Participants believed they should skip doses or not start on ARVs at all if they could not afford the added nutritional burden; 4) Competing demands between costs of food and medical expenses led people either to default from treatment, or to give up food and wages to get medications; 5) While working for food for long days in the fields, participants sometimes forgot medication doses. Despite these obstacles, many participants still reported high ARV adherence and exceptional motivation to continue therapy.

**Conclusions:**

While reports from sub-Saharan Africa show excellent adherence to ARVs, concerns remain that these successes are not sustainable in the presence of widespread poverty and food insecurity. We provide further evidence on how food insecurity can compromise sustained ARV therapy in a resource-limited setting. Addressing food insecurity as part of emerging ARV treatment programs is critical for their long-term success.

## Introduction

Non-adherence to antiretroviral (ARV) therapy is one of the most important predictors of incomplete HIV RNA suppression, immunologic decline, progression to AIDS and death [1], [2], [3], [4], [5], [6]. ARV non-adherence creates particular challenges in resource-limited settings. Stopping ARV therapy for two or more weeks can lead to drug-resistant virus and negate the clinical benefit of the only medications currently available in settings with few treatment options.

When highly active antiretroviral therapy (HAART) was first introduced in sub-Saharan Africa a decade ago, the medications were generally sold to patients at prohibitive prices. These expenses were among the most significant barriers to ARV treatment adherence [7], [8], [9], [10], [11]. In recent years, international aid programs, such as the Global Fund to Fight AIDS, Tuberculosis, and Malaria and the U.S. government's President's Plan for Emergency AIDS Relief (PEPFAR), have substantially expanded support for programs that provide ARV medications free of charge in sub-Saharan Africa and elsewhere. While these efforts have led to improvements in treatment retention and adherence [12], they have not eliminated all socio-economic and structural barriers to accessing treatment and sustaining a long-term medication regimen. [13], [14], [15].

Food insecurity, defined as “the limited or uncertain availability of nutritionally adequate, safe foods or the inability to acquire personally acceptable foods in socially acceptable ways” [16], has recently been identified as a key structural barrier to ARV adherence and as a contributor to ARV treatment interruptions in resource-poor settings [13], [17], [18], [19], [20]. In a qualitative study from Uganda, Tanzania, and Malawi, hunger during HAART initiation emerged as a leading obstacle to ARV adherence [13]. In a study in Northeastern Uganda, consuming only one meal per day and being dependent on caregivers for food were risk factors for ARV non-adherence [21]. In Zambia, the belief that ARVs must be taken with food led individuals to skip doses when they could not access enough to eat [18]. Lack of food was also among the key barriers to ARV adherence in a qualitative study from South Africa [20].

Uganda has a high prevalence of both food insecurity and HIV/AIDS and is an appropriate environment to explore the overlap between these two epidemics. In Uganda, AIDS is responsible for up to 12% of annual deaths and has surpassed malaria and other conditions as the leading cause of mortality among individuals between the ages of 15 and 49 [22]. Food insecurity is also widespread. In a study among PLWA living in urban areas in Uganda, 95% of households reported that they sometimes or often had to eat less preferred foods, 62% reported that sometimes or often all household members had to skip meals, and 22% reported that sometimes or often all household members did not eat for an entire day [23].

While the World Health Organization, UNAIDS, and the World Food Program have recommended integration of food assistance into HIV AIDS programming, [24], [25], [26], [27], [28] there has been little research on the mechanisms through which food insecurity may lead to gaps in treatment and compromise ARV effectiveness. Understanding such mechanisms is important for designing ARV treatment programs that incorporate food or nutritional supplementation and guiding policy decisions about intervention strategies. The prior qualitative studies that identified links between food insecurity and ARV non-adherence were designed to examine barriers to ARV adherence in general, and did not investigate in depth the multi-faceted relationship between food insecurity and non-adherence. We undertook a qualitative study in Uganda to understand and contextualize the role of food insecurity in the lives of HIV-infected patients and to investigate the pathways and mechanisms through which food insecurity could lead to missed doses and treatment interruptions.

## Methods

In August and September of 2007, we conducted semi-structured interviews with 47 women and men living with HIV/AIDS. Forty-one of the participants—29 women and 18 men– were selected from among those arriving each weekday morning to pick up their monthly supply of ARVs or to attend to other health care needs at the Immune Suppression Clinic at the Mbarara University of Science and Technology in Mbarara, Uganda, a trading center for the country's southwest region. The remaining six were selected from patients on early self-pay ARV therapy from the Adherence Monitoring Ugandan Cohort Study in Kampala, Uganda.

Participants were eligible for inclusion in the study if they were HIV-infected adults 18 years or older receiving HIV care at one of the study sites. We sampled using an inductive and iterative approach, while seeking to capture diverse perspectives and characteristics. We aimed to interview both women and men, and individuals on both free and self-pay ARVs, as well as some who had not yet initiated ARV therapy, in order to study barriers to ARV initiation. We also sought participants from both urban and rural settings. Sample size was determined by theoretical saturation, the point at which new categories and dimensions no longer emerged from interviews. Clinic staff familiar with the histories of many attendees helped to identify and recruit potential participants in the study. Written informed consent was obtained from all participants by an experienced local interpreter, who was also a trained interviewer. All interviews were conducted in a private location at the clinic. Patients who agreed to participate in the study were compensated for their time with reimbursement for transportation costs to and from the clinic. All study procedures were approved by the Committee on Human Research at the University of California, San Francisco, the Mbarara University of Science and Technology (MUST) Institutional Review Board (Mbarara, Uganda), the Makarere University Faculty of Medicine and the Uganda National Council for Science and Technology (Kampala, Uganda).

### Data collection

Most interviews from Mbarara were conducted in English and Runyankole, and those from Kampala in English and Luganda, the key languages spoken in the respective regions. One of the study investigators conducted most interviews in conjunction with a trained interviewer and interpreter. Interviews lasted from 30 minutes to 2 hours.

The interview guide included semi-structured and open-ended questions, followed by probes based on initial responses. This approach ensured that specific topics were covered in each interview, but also allowed for the emergence of new and unexpected themes. Our probes were designed to elicit detailed descriptions of participants' opinions, behaviors, and survival strategies in the domains of food security and ARV adherence. All participants were asked about their overall food situation, how often they were hungry, and where and how they found food to feed themselves and their families. If they were already taking ARVs, they were asked about general barriers to ARV adherence, and more specifically whether their food situation impacted their ability to take their medications as prescribed. Where relevant, participants were asked to describe in detail the ways in which food insecurity might pose a barrier to ARV adherence. For participants not yet on ARVs, we investigated the reasons that they had not yet initiated HAART, including any reasons related to food, and key barriers to initiating ARV treatment in the future.

### Data Analysis

Interviews were digitally recorded, translated into English, and transcribed verbatim. The study interviewer maintained field notes on contextual details and impressions that were not captured in the interview transcripts. We analyzed the data by relying on the strategy of grounded theory using open and axial coding [29]. This process involved reviewing, comparing, labeling, and categorizing the data, and then drawing connections between categories according to a coding strategy that involved conditions, context, interactions and consequences. Two of the study investigators conducted an initial review of the interviews and identified repeated observations in the data, collating those into major themes and related sub-themes. Themes and subthemes were revised and refined by successive returns to the data. Peer debriefing was conducted to ensure trustworthiness of the data and to assist in refining the coding strategy.

## Results

The participants ranged in age from 24 to 59 years, with a median age of 37 for the women and 40 for the men. Of the Mbarara participants, 23 were from rural areas, 12 from peri-urban areas, and six from urban areas. Of the Kampala participants, five were from urban areas and one was from a peri-urban area. Small-scale farming, which many refer to as ‘gardening,’ was the most frequent occupation, although many also reported earning income from selling food, clothes, and other basic goods. Thirty-six reported being on a HAART regimen for time periods ranging from one month to several years. Eleven had not yet initiated HAART.

### Food insecurity among participants

Food insecurity was common among participants, whether or not they reported full adherence to their ARV regimens. All but two reported having to struggle to find enough food to feed themselves and their families. Women and men both experienced significant food insecurity, but men were at times favored in terms of food distribution within the household. As explained by one HIV-positive widow: “Before you get married, your parents tell you that you're supposed to feed your husband, that he must eat more food. So when I got to my husband's home, whether I was sick or anything, he must have more food according to what I was told.” Women were also more likely than male respondents to report being separated or widowed, which often reduced their access to food. This was especially the case if they were the sole or primary provider not only for themselves but for children, aging parents, ailing siblings, orphaned nieces and nephews, and other relatives impacted by AIDS. Women who remained with their husbands or partners sometimes reported that fear of loss of access to food for themselves and their children constrained their freedom of action.

Both men and women reported that they could often only afford to eat once or twice a day. Not surprisingly, food insecurity was associated with persistent or intermittent hunger. According to a widowed mother of four, hunger is “almost a daily occurrence for me, I wake up and eat leftovers from last night, and then I'll go work in someone's garden. But that person won't give me lunch, and I'm supposed to work there the whole day.” Her experience, she added, was common in her rural district: “There are 50 families in my village; only five can afford to have food all the time, so the rest are looking for food from those families.”

Even many of those with plots of land for gardening reported that they could not plant and harvest enough to avoid hunger. Both men and women reported that they would feed their children before taking food themselves when food was scarce. A 57-year-old woman, who was responsible for feeding three grandchildren as well as her two youngest children, explained: “I eat less food so my children can eat, because their lives are ahead of them, and mine is about to end, and they feel the privation of hunger more than I do. So I eat less.”

In addition to not having enough to eat, many participants reported being able to access only poor quality food and a limited selection of items. According to a 30-year-old widow who sold goods door-to-door:

I eat matoke, beans, sweet potato, and posho. Sometimes I like it, but most of the time, I feel like I should change what I'm eating, but I can't afford to…. from Monday to Friday we're eating the same thing all the time. That's all we have.

Many participants described eating fish, poultry, and beef only a few times a year, at most. They also reported that poor quality diets and hunger compromised their physical and mental well-being. As stated by a 44-year-old woman with no work: “If I could eat what I'd like to eat, I wouldn't be on edge all the time about my life.”

### Food insecurity and ARV adherence

While many participants were motivated to take their ARV medications as prescribed, 14 out of the 36 patients on ARVs acknowledged that they had missed doses. Food insecurity emerged as one of the most significant barriers to antiretroviral adherence. This was the case both for participants who had missed doses as well as for some who had so far managed to stay adherent but were concerned about their ability to sustain high levels of adherence in the future in the face of significant food insecurity. The impact of food insecurity on adherence was particularly salient among women in our sample. As stated by a 42-year-old woman who reported severe stomach pain if she took her ARVs without food: “In a week when I missed, that is when I have not gotten food to eat. What happens sometimes I have to force myself to take the medicine, but other times I cannot force myself to take the medicine, so I just leave it.” A widow whose husband died of AIDS and who began treatment in 2005 said that she skipped some doses altogether and delayed other doses when she couldn't find enough food:

The most difficult or painful thing that I find about taking my medicine is when the time for taking medicine approaches and I don't have something to eat. If I don't have something to eat, then I don't take my medicine until I have what to eat.

In some instances, participants suggested that ARV adherence could lead to greater food security because of improved health and ability to work; conversely, ARV non-adherence, by causing a decline in health, could lead to greater difficulties in accessing food. An older woman who had started ARVs two years previously explained how the treatment had helped her in her struggle for provisions: “These days it's much easier to do the gardening. Before I started my treatment, I was really, really sick, but since I started I'm much stronger, I can carry stuff around, I can go to garden and weed, and I am able to do much more.”

During the interviews, five mechanisms emerged through which food insecurity undermined ARV adherence among both men and women as well as impacted decisions about ARV treatment initiation: 1) ARVs often increased appetite, and food scarcities exacerbated the resulting hunger; 2) Side effects of ARVs were reported to be much worse in the absence of food; 3) Participants were counseled on the importance of taking ARVs with a balanced diet and reported that some people did not start ARVs because they could not afford the accompanying food; 4) Competing demands between the cost of obtaining food and the cost of medical treatment and transportation for monthly clinic visits led people either to default from treatment, or to give up food and wages to get medications; and 5) Long days of farming in the fields or doing other work to earn money for food sometimes caused people to skip or forget to take their doses. These varied mechanisms led to missed doses and reports of delays in treatment initiation as well as early treatment discontinuation. We also explored the role of food supplementation on food insecurity and adherence.

### 1. Increased appetite with ART

In many cases, participants reported that ARVs significantly increased their appetite, which caused difficulties for those already struggling to feed themselves and their families. As stated by a 44-year old widow and mother of four: “Sometimes [after taking ARVs] I am so hungry, it just comes and it's intense, my whole body is shivering from hunger.” According to a 26-year-old woman with two children:

The ARVs made me hungrier…like you want to eat all the time, even sometimes you feel that before you take them, there should be something to eat. And two hours after taking ARVs, you're very hungry and feel like taking something.

A widow with six children compared the intense hunger associated with taking ARVs to that of pregnancy: “It's like when you're pregnant, you crave a lot of things. But even when you crave things, you can't have them.”

Many participants reported living meal-to-meal rather than having the resources to afford to keep much food at home. They often had developed carefully calibrated eating strategies to make food supplies last. The additional hunger associated with taking ARVs threatened to disrupt the fragile stability of these strategies. A 35-year-old mother of three, whose husband died of AIDS in 2001, explained:

Sometimes when we swallow this medicine we feel like eating all the time. So you eat and feel satisfied but in a few minutes, you feel hungry and you want to eat again, and yet you can have prepared very little food that you have eaten and finished…maybe a few fingers of matoke, a quarter kilogram of rice, and therefore you just stay hungry without anything to eat until in the evening when you have to go and buy another quarter [kg.] of rice.

While many reported difficulties associated with hunger from taking ARVs, in some cases this hunger contributed to non-adherence. For example, a 42-year-old mother of four explained that she sometimes skipped doses to avoid the pain of feeling hungry:

Sometimes you could have had a bad breakfast, you didn't have any lunch, and supper is also not good, and so you really don't feel like taking the medicine, so you don't take it. Because if you take it, you feel very bad in your stomach, very, very bad. So, so hungry like never before, and you're trembling, and the daylight is not coming soon enough. You feel shaking, you're feeling this very bad hunger, you can't even sleep.

Some patients not yet on ARVs expressed concern about the prospect of increased hunger. A 34-year-old mother of four said her sister-in-law reported being “hungry most of the time” after starting on HAART. The woman herself was not yet taking ARVs but her husband was about to start. The prospect of her husband's hunger in the absence of adequate food was a significant source of stress in her life:

I am worried about my husband's situation and we have discussed it, which is why today we went to the counselor together to find out whether they could reduce the quantity or give us medicine that's not as strong. Because if my husband is hungry all the time and there's no food, then what happens?

### 2. Worse ARV side effects

Many participants reported that taking the ARV medications without sufficient food exacerbated medication side effects. In addition to increased hunger on ARVs, these reported side effects included headaches, stomach pain, dizziness, shivers or tremors, loss of energy, fainting, sweating, and rapid heartbeat. “When you are on ARVs, when you take it on an empty stomach, you don't feel well,” said a 50-year-old widow. “You feel like you have ulcers, like pain in the stomach.” A young woman who reported feeling hungry at least twice a week noted: “Every time I take the medicine without eating anything, it does me bad, it saps my energy. So I struggle to make sure before I take my medicine, I look for something.” A 39-year-old married man with three children reported a wide range of symptoms:

When I take my ARVs when I haven't eaten or there's no food to eat, I feel uncomfortable, and also I'm shivering, and sweating and might pass out. What I usually do when I don't have food is take the medicine and then go cover myself up in bed. And my heart rate increases.

Similarly, a 34-year-old mother of five, whose husband died of AIDS in 2002, described the difficulties of taking ARVs without food:

When I have to take my medication and I have not eaten, I get stomach aches and I feel like my heart has been misplaced— plucked out from it's normal place—and then I get a runny stomach. And I get diseases or infections that I didn't have when I take it [the medication] with food.

Not surprisingly, many also reported the converse: they experienced no side effects when they took the medications after having eaten a sufficient amount. For instance, a woman who suffered from intense hunger, trembling, and insomnia when she took ARVs without food described the absence of adverse effects when she took them on a full stomach: “This medicine, before you take it if you're really full, you have had a good breakfast, a good lunch, and then also had a good supper, then you take them and there are no side effects.”

Despite side effects, many participants were strongly motivated to take their ARVs at the prescribed times even when sufficient food was not available. For example, a woman who had been in treatment for four years continued to struggle with the side effects of taking ARVs without food: “Most of the time when I swallow the medicine before I've had food, I feel dizzy in my eyes. I also get pains in my stomach, like slashing pains in my stomach when I take medicine without food.” Despite this, she did everything possible to ensure that she adhered to her medication regimen: “When it comes for time to taking medicine, even if don't have the food, I must take it, because if I don't, I will be disorganizing something within my life system.”

A 34-year-old woman, who was separated from her husband and was living with her parents, said the problem of more severe side effects of ARVs in the absence of food was a frequent topic of conversation among patients, but that most accepted this downside because they recognized the benefits:

A lot of people are always complaining about having to take the medication with nothing, when they have not eaten anything. But since it's also for helping them, they decide to take it even when they know it's going to affect them and make their stomachs ache, because they don't have much of a choice.

### 3. Advice to take ARVs with a balanced diet

Participants reported that before starting on medication they had heard–through word-of-mouth from family members, friends, other patients, or counselors– about the need to eat a balanced diet on ARVs. Some of what they heard made ARV-naïve participants question whether they would be able to sustain the regimen or their health. A 24-year-old woman, pregnant with her second child, said she hoped to start taking ARVs soon, but was concerned about what would happen if she initiated treatment without adequate nutritional support:

What I am worried about most is the fact that they say when you're on ARVs, you need to drink a lot and eat very well, but I don't think I can afford that…They said it's a very strong medicine and if you don't drink a lot and eat a lot, then your energy is sapped very fast, and you deteriorate very fast.

As a result of these concerns about perceived nutritional demands, participants reported that some people who might otherwise have initiated HAART chose not to. For example, a 30-year-widow, who worked as a *hawker*—someone who sells merchandise door-to-door—said food insecurity could lead directly to delayed ARV initiation or a decision not to seek medical care altogether:

Those who are sick fear to come for treatment, because they know if they come and their CD4 count is low, doctors will ask them to eat a balanced diet. But then they say they cannot afford the food and the balanced diet. To afford such food is very expensive. So they think they can't afford it, and they don't even come for treatment. Since taking ARVs according to what they've heard necessitates you to spend more money on food.

In addition to delaying HAART initiation, some individuals stopped taking their ARVs altogether when they thought that they could not follow the advice of health care providers to eat a balanced diet. A widowed mother of three, aged 35 years, reported that a friend had decided to stop taking her ARVs because she could not sustain the nutritional demands of ARVs:

She called me on Friday and said she can't handle what comes with the medicine, it's draining her. According to what they are telling us here about positive living, my friend feels that she will not be able to do the positive living, since taking the medicine comes with a change in diet that she cannot afford, since she is renting a house. So according to her budget, she feels that stopping the medication would be the best thing.

### 4. Competing demands

Another theme expressed by participants was the need to make the difficult choice between spending limited resources on medical needs—such as the cost of transportation to clinic or the cost of additional medications to prevent opportunistic infections—and spending it on food for themselves and their families. Participants had to either sacrifice an adequate diet for themselves and their children to afford transport to clinic or other health-related expenses, or they had to forego picking up their ARVs in order to access something to eat. Demands for food and health-related expenses also competed with other essential needs such as school fees for children, clothing, and household expenses, compounding the difficult choices families had to make. Some married women expressed constraints in their ability to make these economic decisions because of differences with their husbands, who sometimes spent money just on themselves or had second families to support. At the same time, women who were separated, divorced or widowed often reported having to stretch their provisions to feed a large extended family, reducing the amount of funds available for medical needs. These impossible choices caused significant anxiety for many individuals in our study; in some cases, they also contributed to ARV non-adherence, treatment interruptions, and fears about HAART initiation.

A 34-year-old woman reported her regret at having to spend 500 shillings ($0.25) for a boda-boda [motor-scooter taxi] to clinic when she had nothing at home to eat. “If I had something to eat at home, I wouldn't feel bad, because the 500 is also helping me to come here.”

A 35-year-old widow said she has missed some appointments with the doctor because food costs for her and her three children absorbed the money she needed to pay for transportation to the clinic. “What happens is that all the money you get you have to eat, because you buy water, you buy everything,” she said. “By the time it comes to the clinic, you don't have the money.” She added, however, that she always found a way to come when it was time to pick up her monthly supply of medication no matter what her food situation, although she recognized that travel to clinic entailed significant opportunity costs:

Sometimes, when I am about to go back home and I have not left anything in the house to eat, I ask myself when I get back home, what will I eat? That's when I feel I have wasted time by coming to the clinic. But then if I didn't come to the clinic, I wouldn't be able to get the medicine.

Participants expressed considerable anxiety over having to make such difficult choices. A 32-year-old married mother of five reported once missing weeks of medication because she didn't have the money for both clinic-related expenses and food for her children:

Sometimes there is stress between my medical needs and my food needs, because I have to spend a lot on food, but for medication things I must spend on them, and I find I am spending a lot on food, and it stresses me. I spend on transport, but also the multi-vitamins, I have to supplement them because they give us just a little.

Individuals also often compromised the quality of their diet to afford medical expenditures. As a result, ARVs increased food insecurity in the short term for some participants when limited resources were spent on medical care instead of food.

### 5. Working for food

Many participants reported that their efforts to access food—whether through farming for long days in the fields or spending hours seeking paid work to buy something to eat— was a barrier to ARV adherence. For example, the 30-year-old hawker who had been on ARVs for four years described how working for food could interfere with taking doses as prescribed:

The problem I see is that once in a while I forget to take my medicine at the allocated time…I have to work for food and thus by the time it comes for taking my medicine I am maybe too busy with work and I have forgotten about my medicine…Sometimes you wake up and don't have posho in the house to make porridge, and where you have to buy it from is very far and you don't have the money, but by the time you get the money and get the posho, it's late.

A 57-year-old widow, who earned money selling food and bricks, said she missed her morning dose on days when she could not find food. “Not taking my doses for morning, it happens about twice a week, and I'm looking for food during that time,” she said. “So that by the time it comes to 7 pm [time for her night dose] I have found food, so I am able to take my dose.”

A number of participants reported having been told not to take their ARVs if they missed their appointed dose time by an hour or two. As a result, some said they missed doses because by the time they could stop working or take a break in their search for food to eat, they were already more than an hour or two late. A 40-year-old father of five and farmer whose wife also had HIV said that he never missed his night dose, but added:

For the morning dose, sometimes I've gotten a small job, and while I'm working, I realize it's an hour late or an hour and a half late. And we are sensitized that we shouldn't take our medication past a certain time. So I miss that dose maybe twice a month.

A 38-year-old farmer and food seller not yet on ARVs expressed concern that he'd be too busy working all day to have time to eat and drink the amount of food and liquid required with ARVs. “I find myself worrying about what would happen to my health if I did not take the medicine for a whole day as prescribed, since I have to go to work and as such cannot eat and drink as much as prescribed.”

### Food insecurity and food supplementation

None of the participants reported receiving any supplemental food provisions at the time of the interview, although one mentioned having received such assistance in the past.

As a result of the strong link between food insecurity and non-adherence, participants emphasized the importance of food supplementation as part of comprehensive HIV/AIDS care. As explained by the 57-year-old widow:

If they could distribute a little maize flour with the medicine, so that the person has something to take their medicine with, it would be much easier. Most of people who are on ARVs are not able to look after themselves or buy food, so most of them come when they are almost at death point, so what they do is start them on ARVs when they don't have food…, and the ARVs kill them because they have no food…But if they came and got posho alongside ARVs, maybe they'd have something to drink or eat when they start their medication.

This participant added that once participants have been on ARVs for some time, the food support could stop because “ these people have already regained energy to start supporting themselves.”

## Discussion

This study is the first to our knowledge to investigate the potential mechanisms through which food insecurity interferes with ARV medication-taking behavior. We found that food insecurity and hunger not only interfere with day-to-day adherence but that fears about hunger and food insecurity may also cause people to delay initiating or to discontinue ARV therapy. Preventing hunger and malnutrition are important goals in their own right and should comprise a critical component of comprehensive health care for HIV-infected patients. Since food insecurity may be contributing to delayed ARV initiation, incomplete adherence, and ARV treatment discontinuation, food and nutrition interventions may be an important adjunct to the substantial investment in ARV programs in resource-limited setting.

We found five mechanisms through which food insecurity impacted ARV adherence: increased hunger with ARVs, worse ARV side effects in the absence of food, counseling on the need to take ARVs with food, competing demands between food costs and health care expenses, and forgetting or being unable to take ARV doses while working for or searching for food. Several of these mechanisms have been reported in other studies from sub-Saharan Africa [17], [30], [31].

While the mechanisms for how food insecurity impacted adherence were generally similar among women and men, women were at times less favored in terms of household food distribution. In addition, women—especially those who were widowed or otherwise on their own–often reported being solely responsible for feeding children and other family members. As a result, the impacts of food insecurity on treatment non-adherence were more salient among women. Gender disparities in the experience and health consequences of food insecurity have also been reported in other settings in sub-Saharan Africa [32], [33]. Taken together, these findings highlight the strong linkages between food insecurity, gender inequality and worse health outcomes and suggest that gender inequality and discrimination should be critical targets for HIV/AIDS programming.

Despite the substantial obstacles to ARV adherence, motivation to adhere was extremely high in our study, consistent with previous studies. [7], [12], [34] However, people living with HIV/AIDS may not be able to maintain high levels of adherence over the long-term in the face of such obstacles as experiencing intractable hunger on HAART, or having to choose between feeding their families and paying for health-related expenses. Moreover, treatment options are limited in many resource-constrained settings; if resistance to first-line drugs develops because of poor adherence, the negative consequences are more significant than in countries with access to a broader range of medications. Limited treatment options also makes it harder to address ARV side effects related to food insecurity, since alternative regimens with different side effect profiles are less readily available.

In addition to the negative consequences of food insecurity on day-to-day ARV adherence, our study suggests that food insecurity is a risk factor for ARV treatment interruptions and discontinuation, both which have been shown to be associated with virologic failure, worse clinical outcomes and mortality. [35], [36], [37] Moreover, early ARV initiation has been associated with decreased mortality [38], [39], and our data also show that food insecurity is a risk factor for delayed treatment initiation. Previous data has directly linked food insecurity with incomplete HIV RNA suppression and mortality even when controlling for effects of adherence [40], [41]. It is possible that these associations between food insecurity and poor clinical outcomes are mediated in part through impacts of food insecurity on delayed HAART initiation and treatment discontinuation, although more studies are needed to assess this possibility.

The negative impacts of food insecurity on ARV adherence, treatment continuation, HIV RNA suppression, and mortality argue for the need to incorporate food supplementation and sustainable food production strategies as essential components of emerging ARV treatment programs. Nutritional supplementation can directly improve both ARV adherence and retention in care. In an ecological analysis of programmatic factors associated with loss to follow-up in eight countries in sub-Saharan Africa with over 100,000 individuals, Nash et al. [42] found that programs that offered nutritional support had substantially lower rates of loss-to-follow-up compared with programs that did not. Similarly, in a pilot intervention study in Zambia, individuals receiving food supplementation with HAART achieved significantly higher ARV adherence than individuals not receiving food supplementation [43].

Many local organizations in Uganda have recognized that adequate food and nutrition are critical to an effective response to the HIV/AIDS epidemic, and some have initiated programs to improve food security among HIV-infected individuals. [44] The AIDS Support Organization (TASO) is the largest non-governmental organization (NGO) providing AIDS prevention and care services in Uganda and has been collaborating with other NGOs to expand nutritional support to PLWA. There is also a food supplementation program for severely malnourished patients hospitalized on the medical wards at Mbarara University of Science and Technology.

In addition to basic health training and direct food supplementation, other interventions that are being scaled up by local NGOs to improve the food security of PLWA include income generation activities through small enterprises, home and community gardening activities, and support for animal husbandry and crop production. [44]

### Limitations

There were several limitations to our study. We did not interview individuals who were not yet linked to care nor individuals who were lost to follow-up; it is possible that food insecurity poses an even greater barrier to HIV care for members of these two groups and that our data therefore understate the actual impact of food insecurity on ARV treatment outcomes. Since interviews were conducted among individuals attending clinic and consenting to study procedures, the high levels of adherence reported by participants may be an overestimate of actual ARV adherence in this setting. In addition, the majority of participants interviewed were from rural Uganda, with only a few participants from an urban clinic in Kampala. We were therefore unable to draw significant distinctions between urban and rural areas in the mechanisms through which food insecurity may interact with adherence. We chose not to carry out focus group discussions because of confidentiality concerns and, especially, the sensitive nature of the subject matter discussed. Due to time constraints, no participant observation was conducted . Future studies on this topic would benefit from more diverse qualitative techniques to help triangulate the findings.

### Conclusion

In this qualitative study from Uganda, we found that many participants suffered from daily hunger, and that food insecurity contributed to ARV non-adherence, delayed HAART initiation, and treatment discontinuation. Freedom from hunger is a basic human right and warrants immediate attention in its own right. The link between food insecurity and poor ARV outcomes further heightens the importance of addressing food insecurity as part of comprehensive care among HIV-infected individuals worldwide.
